# Highly purified-hMG *versus* rFSH in ovarian hyperstimulation in women undergoing elective fertility preservation: a retrospective cohort study

**DOI:** 10.5935/1518-0557.20240099

**Published:** 2025

**Authors:** Tal Israeli, Nivin Samara, Shimi Barda, Asnat Groutz, Foad Azem, Hadar Amir

**Affiliations:** 1 Racine IVF Unit, Fertility Institute, Lis Maternity Hospital, Tel Aviv Sourasky Medical Center, Tel Aviv, Israel affiliated to the Faculty of Medical & Health Sciences, Tel Aviv University, Tel Aviv, Israel

**Keywords:** fertility preservation, ovarian stimulation, rFSH, hp-hMG, ovarian response

## Abstract

**Objective:**

To compare recombinant FSH (rFSH) with highly purified-human menopausal gonadotrophin (hphMG) on ovarian response in women undergoing elective fertility preservation (FP).

**Methods:**

This retrospective study included 456 women who underwent elective FP with gonadotropinreleasing hormone (GnRH) antagonist or progestin-primed ovarian stimulation (PPOS) protocols between 01/201712/2021. Only the first treatment cycle of each woman was included. 341 women were stimulated with rFSH and 115 with hp-hMG, and the ovarian stimulation outcomes were compared. A multivariate linear regression assessed the impact of age, basal FSH, antral follicle count (AFC) and protocol and gonadotropin types on the outcomes.

**Results:**

Women in the rFSH group were significantly younger, and their AFC was significantly higher than those in the hp-hMG group (35.50±2.12 *vs*. 35.99±2.13years, *p*=0.034 and 13.76±6.08 *vs*. 11.84±6.06, *p*=0.002). There were no significant group differences in the amount (*p*=0.645) and duration (*p*=0.265) of FSH stimulation. The estradiol level was significantly lower for the rFSH group compared to the hp-hMG group (2547.18±1648.21pg/ mL *vs*. 3468.02±2497.69pg/mL, *p*<0.001), while the progesterone level was significantly higher (1.33±0.75 ng/mL *vs*. 1.01±0.52ng/mL, *p*=0.001). The numbers of retrieved and MII oocytes were significantly higher for the rFSH group compared with the hp-hMG group (16.82±10.95 *vs*. 13.25±9.66, *p*=0.02, and 13.22±9.13 *vs*. 9.76±7.11, *p*=0.005), while the maturity rates were comparable (p=0.103).

**Conclusions:**

Patients in the rFSH group had higher numbers of both retrieved and MII oocytes when undergoing elective FP.

## INTRODUCTION

Recombinant follicle stimulating hormone (rFSH) and highly purified-human menopausal gonadotrophin (hphMG) are widely and successfully used for ovarian stimulation in infertile women undergoing assisted reproductive technology (ART) procedures. hp-hMG is a mixture of FSH, luteinizing hormone (LH), and human chorionic gonadotropin (hCG with LH-like properties) that are collected, extracted, and purified from the urine of post-menopausal women. The usual preparation contains a 1:1 ratio of FSH and LH (e.g., 75 IU each) (Wolfenson *et al*., 2005). rFSH is manufactured by recombinant DNA technology using Chinese hamster ovary cell lines transfected with the genes encoding for the two human FSH subunits or genes derived from a cell line of human fetal retinal origin. rFSH is completely free of any LH (Out *et al*., 1995; Olsson *et al*., 2014).

Many studies have compared the outcome of rFSH and hMG for ovarian stimulation, with most having been conducted on infertile women undergoing pituitary down-regulation with a gonadotropin-releasing hormone (GnRH) agonist long protocol. Two meta-analyses and one Cochrane review showed a better outcome in terms of the live birth rate when hp-hMG was used compared with rFSH in the GnRH agonist long protocol (Al-Inany *et al*., 2008; Coomarasamy *et al*., 2008; van Wely *et al*., 2012). A recent systemic review and meta-analysis supported that conclusion but found the evidence of a difference in the cumulative live birth rate to be insufficient (Bordewijk *et al*., 2019). One of the most popular protocols currently employed for treating women undergoing ART utilizes the GnRH antagonist. GnRH antagonists induce a rapid decrease in LH and FSH levels, thereby preventing spontaneous LH surges. Previous studies have compared the effectiveness of rFSH with that of hp-hMG in infertile women undergoing in-vitro fertilization (IVF) by means of an antagonist protocol and found no benefit of one over the other (Bosch *et al*., 2008; Requena *et al*., 2010; Ana *et al*., 2011; Devroey *et al*., 2012; Shavit *et al*., 2016). Progestin-primed ovarian stimulation (PPOS) is new ovarian stimulation protocol that was recently proposed to achieve multi-follicle recruitment in women undergoing IVF. It uses progesterone instead of the traditional down-regulation with a GnRH antagonist to block the LH surge (Ata *et al*., 2021). There are no published studies that compare ovarian stimulation outcomes of hp-hMG with r-FSH in women treated with the PPOS protocol.

All of the available studies that compare rFSH with hp-hMG have been performed in infertile couples. The pathogenesis underlying the etiology for infertility may be relevant when examining the effect of various gonadotropins with different biological activity. For example, both the quantity and quality of oocytes are markedly diminished among women with endometriosis or advanced age compared to those in healthy controls (Oral *et al*., 2015). Some researchers have reported improved oocyte/embryo quality after exposure to exogenous LH activity (Lisi *et al*., 2005; Ziebe *et al*., 2007). Others have proposed that LH activity appears to be relevant in IVF patients, mainly when the cause lies in the female factor or unexplained infertility rather than the male factor (Platteau *et al*., 2004). It is also arguable that one potential reason for the inconsistency of the results is such different etiologies of infertility. These considerations taken together; we were interested in comparing the effects of rFSH with hp-hMG on ovarian response in women who apparently do not have a fertility problem.

A significant portion of the patients in IVF units are women who undergo fertility preservation (FP). FP is carried out for both medically indicated and elective reasons. The former includes young women with low ovarian reserve or those at risk for early ovarian failure (cancer patients, genetic conditions, severe endometriosis, unexplained background, family background) and the latter is generally age-related (Cobo *et al*., 2013). It is well known that advanced maternal age (>35 years) (Dunson *et al*., 2004; Sozou & Hartshorne, 2012) is associated with a decline in both follicular pool number and oocyte quality, as well as posing higher risks of fetal chromosomal abnormalities that result in fetal loss, representing the leading causes of age-related fertility decline (Hook, 1981; Faddy *et al*., 1992; Nybo Andersen *et al*., 2000). Currently, the proportion of women delaying childbearing until the late 3^rd^ to early 4^th^ decade of life has greatly increased, especially in Western societies (Mills *et al*., 2011; Schmidt *et al*., 2012). Oocyte cryopreservation affords women the chance to conceive and deliver their genetic offspring at a future date (Cobo *et al*., 2013). These are characteristically healthy, ostensibly fertile women who wish to postpone motherhood for various reasons, such as educational or career ambitions, or because they had not yet found a partner (Hodes-Wertz *et al*., 2013; Nasab *et al*., 2020). Women undergoing elective FP are at a higher risk of ovarian hyperstimulation syndrome (OHSS). A major advantage of using GnRH antagonist and PPOS protocols in women who are considered fertile is the possibility of preventing OHSS (Al-Inany *et al*., 2016; Guan *et al*., 2021) by triggering ovulation with a GnRH agonist (Youssef *et al*., 2014; Kalafat *et al*., 2022).

The present study aimed to compare the ovarian stimulation outcomes of hp-hMG versus r-FSH in women undergoing elective FP treatment by means of the GnRH antagonist and PPOS protocols.

## MATERIALS AND METHODS

### Ethical approval

The study processes are in accordance with the ethics standards defined by the committee in charge for studies in humans (the ethics committee of the Tel Aviv Sourasky Medical Center) and in accordance with the Helsinki declaration (#0325-22-TLV). Informed consent was waived for this retrospective and anonymous analysis.

### Study population and participant recruitment

**Figure 1:**
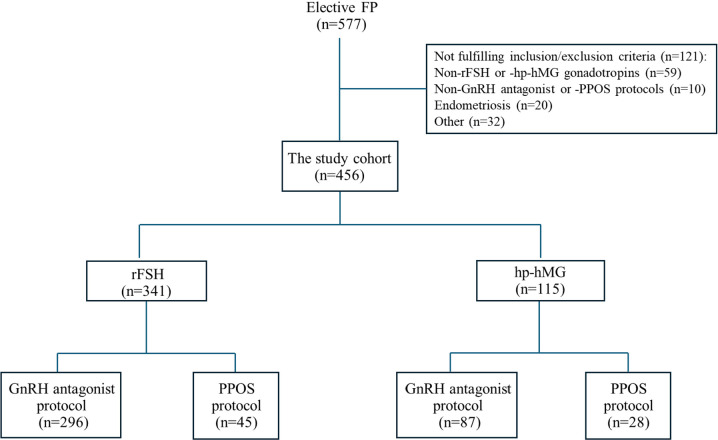
Study flow chart. FP, fertility preservation; rFSH, recombinant follicle-stimulating hormone; hphMG, highly purified-human menopausal gonadotropin; GnRH, gonadotropin-releasing hormone; PPOS, progestin-primed ovarian stimulation.

This retrospective study was performed between January 2017 and December 2021 at the IVF Unit, Fertility Institute, Tel Aviv Sourasky Medical Center, a tertiary university-affiliated medical center. Five-hundred seventy-seven women who underwent their first elective (non-medical) FP treatment were recruited to this study, of which 456 women fulfilled the inclusion criteria and formed the study group (Figure 1). Notably, only women who did not undergo any prior treatments were included in the study. The study did not include women with previous failed attempts of ovarian stimulation or oocyte retrieval. Exclusion criteria: 1. Ovarian stimulation with gonadotropins other than rFSH or hp-hMG; 2. Control of ovarian stimulation with other than GnRH antagonist or PPOS protocols 3. In order to prevent any influence of factors that could affect the outcome of oocyte yield, we also excluded women who presented with the following: endometriosis, a history of ovary surgery, diabetes type 1 and type 2, autoimmune disease (rheumatic arthritis, ulcerative colitis, Crohn’s disease, multiple sclerosis), and being a Fragile X carrier.

### Data collection

All relevant data were collected from the computerized database of the hospital. The data in the electronic charts included the following: clinical details [age, body mass index (BMI), marital status, number of children, thyroid-stimulating hormone (TSH) levels, and prolactin levels], fertility potential markers [basal FSH, antral follicle count (AFC)], ovarian stimulation details (protocol type, ovarian stimulation duration, total FSH dose, and type of ovulation trigger), and outcomes [peak serum estradiol (E2), peak serum progesterone, number of retrieved oocytes, number of metaphase II (MII) oocytes], and maturation rate (derived from the number of MII oocytes/number of aspirated oocytes)].

### Ovarian stimulation data and outcomes

Controlled ovarian stimulation was carried out by the GnRH antagonist or PPOS protocols. The GnRH antagonist had been the preferred protocol in our unit until March 2021 when PPOS became the primary protocol for FP. The change occurred after it emerged that both protocols were equally effective (Ata *et al*., 2021; Guan *et al*., 2021) while PPOS was less expensive. The PPOS protocol involved pituitary suppression by 30 mg/day dydrogesterone (Duphaston, Abbott Biologicals B.V., Netherlands) started simultaneously with the gonadotrophins from the 2nd day of the menstrual cycle until the trigger day. Either rFSH (Gonal-F, Merck) or hp-hMG (Menopur, Ferring Pharmaceuticals) was used for ovarian stimulation. Ovarian stimulation was carried out with a medication determined by the physician’s preference. Several fertility centers use LH supplements, such as hp-hMG or recombinant LH, more frequently for older patients and/or with lower AFC (Humaidan *et al*., 2004; Bosch *et al*, 2011). This approach is also used by physicians in our clinic. Ovulation was triggered with 0.2 mg of triptorelin (Decapeptyl; Ferring Pharmaceuticals) or 250 mcg of choriogonadotropin α (Ovitrelle; Serono, Geneva, Switzerland), or by a combination of both when at least three follicles achieved a diameter of 18 mm. Ovum pickup was performed 36 hours later, and the embryologists determined the total number of oocytes retrieved and the MII oocytes per cycle. All MII oocytes were cryopreserved.

### Statistical analysis

Data were analyzed with SPSS version 27.0 (SPSS, Inc., Chicago, IL, USA). The data were summarized as mean±standard deviation or number of responders (percentage) according to the variables. Continuous variables between groups were compared with the t test. The effect sizes were calculated by means of Cohen’s D. To overcome the obstacle of the difference in patients’ AFC, ANCOVA was performed. By using ANCOVA, we examined the influence of independent variables on a dependent variable while removing the effect of the covariate factor (AFC). Comparison of ovarian stimulation data and outcomes between patients undergoing stimulation with rFSH or hphMG were made after ANCOVA with AFC as a covariate. A multivariate linear regression analysis was performed to control for age, basal FSH level, AFC, protocol type and gonadotropin type as confounders for the estradiol (E2) level, number of retrieved oocytes, number of MII oocytes and maturity rate. Significance was tested with the t-test, Mann-Whitney U test, χ^2^ test, and Fisher’s exact test as appropriate. A *p* value of <0.05 was considered significant. The *P* value was calculated after log transformation for normal distribution for the following variables: TSH, prolactin, AFC, gonadotrophin (GT) total dose, Peak E2, peak progesterone, the number of oocytes retrieved and the number of MII oocytes. The log transformation was also used for the regression model.

## RESULTS

### Clinical characteristics of the study participants

A total of 456 women who underwent elective FP were included in this analysis, of whom 341 were stimulated with rFSH and 115 with hp-hMG (Figure 1). The clinical characterizations of the entire cohort are detailed in Table 1. The women in the rFSH group were significantly younger than the women in the hp-hMG group (35.50±2.12 years *vs*. 35.99±2.13 years, *p=*0.034) and their AFC was significantly higher (13.76±6.08 *vs*. 11.84±6.06, *p*=0.002).

**Table 1:** Comparison of clinical parameters between patients undergoing ovarian stimulation with rFSH or hp-hMG.

	rFSH (n=341)	hp-hMG (n=115)	*p* value
Age (y)	35.50 (2.12)	35.99 (2.13)	0.034
Weight (kg)	62.52 (11.37)	64.64 (14.07)	0.113
BMI (kg/m^2)^	23.34 (3.91)	23.85 (5.01)	0.354
Children 0 1	338 (99.1) 3 (0.9)	111 (96.5) 4 (3.5)	0.071
Basal FSH (mIU/mL)	8.02 (2.7)	8.67 (3.80)	0.059
^*^TSH (µIU/mL)	1.99 (1.06)	1.86 (1.00)	0.113
^*^Prolactin (mIU/L)	271.90 (154.36)	259.46 (131.15)	0.573
^*^AFC (n)	13.76 (6.08)	11.84 (6.06)	0.002

Values are presented as mean (standard deviation) or number (%). A *p* value of < 0.05 was considered significant.

* The *p* value was calculated after log transformation for normal distribution. rFSH, recombinant follicle-stimulating hormone; hp-hMG, highly purified-human menopausal gonadotropin; BMI, body mass index; FSH, follicle-stimulating hormone; TSH, thyroid-stimulating hormone; AFC, antral follicle count Note: Standard reference ranges: FSH: 1-9.2mIU/mL; TSH: 0.5-4.8µIU/mL; Prolactin: 108.78-557.13mIU/L

### Ovarian stimulation data and outcomes

The GnRH protocol was used in 296 women (86.8%) in the rFSH group and 87 women (75.7%) in the hpHMG group, and the PPOS protocol was used in 45 women (13.2%) in the rFSH group and 28 women (24.3%) in the hp-HMG group (*p*=0.008). No significant differences between the rFSH group compared to the hp-HMG group were found in the total FSH dosage (*p*=0.645) or the duration of gonadotrophins treatment (*p*=0.265). The peak E2 level was significantly lower in the rFSH group compared to the hp-HMG group (2547.18±1648.21 *vs*. 3468.02±2497.69 pg/mL; *p*<0.001), while the peak progesterone level was significantly higher in the rFSH group compared to the hp-hMG group (1.33±0.75 *vs*. 1.01±0.52 ng/mL; *p*=0.001). The numbers of retrieved and of MII oocytes were significantly higher in patients stimulated with rFSH compared to those stimulated with hp-hMG (16.82±10.95 *vs*. 13.25±9.66; *p*=0.02, and 13.22±9.13 *vs*. 9.76±7.11; *p*=0.005), while the oocyte maturity rates were also similar (*p*=0.103) (Table 2). Next, subgroup analysis for age was performed for the entire cohort. Table 3 shows the subgroup analysis results for patients younger than 35 years and patients 35 years or older. Both young and old patients in the rFSH group had better cycle outcomes compared to those in the hp-hMG group in terms of oocytes retrieved and MII oocytes. Maturity rates for both younger and older patients were comparable.

**Table 2:** Comparison of ovarian stimulation data and outcomes between patients undergoing stimulation with rFSH or hp-hMG.

	rFSH (n=341)	hp-hMG (n=115)	p value
Ovarian stimulation protocol GnRH antagonist PPOS	296 (86.8) 45 (13.2)	87 (75.7) 28 (24.3)	0.008
Ovarian stimulation duration (days)	11.33 (1.60)	11.02 (1.46)	0.265
^†^GT total dose (mIU/mL)	3157.36 (919.35)	3263.48 (811.20)	0.645
^†^Peak E2 (pg/mL)	2547.18 (1648.21)	3468.02 (2497.69)	<0.001
^†^Peak P (ng/mL)	1.33 (0.75)	1.01 (0.52)	0.001
Final maturation trigger GnRH agonist hCG hCG+GnRH agonist	332 (97.4) 8 (2.3) 1 (0.3)	105 (91.3) 9 (7.8) 1 (0.9)	0.019
^†^Oocytes retrieved (n)	16.82 (10.95)	13.25 (9.66)	0.02
^†^MII oocytes (n)	13.22 (9.13)	9.76 (7.11)	0.005
Maturity rate (%)	77.98 (16.63)	75.10 (21.22)	0.103

* After ANCOVA with antral follicle count (AFC) as a covariate.

Values are presented as mean (standard deviation) or number (%). A *p* value of <0.05 was considered significant.

† The *p* value was calculated after log transformation for normal distribution.

rFSH, recombinant follicle-stimulating hormone; hp-hMG, highly purified-human menopausal gonadotropin; GnRH, gonadotropin-releasing hormone; PPOS, progestin-primed ovarian stimulation; GT, gonadotropins; E2, estradiol; P, progesterone; hCG, human chorionic gonadotropin; MII, metaphase II.

**Table 3:** Subgroup analysis for younger and older patients.

Variable	rFSH, n = 341			hp-hMG, n = 115			Age<35 rFSH x hp-hMG	Age>35 rFSH x hphMG
	Age<35 n = 172	Age>35 n = 162	p-value	Age<35 n = 49	Age>35 n = 66	p-value		
Age(y)	33.80±1.19	37.24il.29	<0.001	34.06il.23	37.42il.39	<0.001	0.186	0.330
BMI (Kg/m^2^)	23.13i3.87	23.57i3.95	0.417	23.93i5.96	23.79i4.30	0.898	0.357	0.758
Basal FSH (mUI/mL)	7.86±2.50	8.18±2.89	0.297	8.4Ü3.91	8.87 (3.72)	0.550	0.250	0.156
AFC (n)	14.65±6.44	12.87i5.56	0.02	13.80i7.02	10.29i4.69	0.003	0.467	0.001
Ovarian stimulation duration (days)	11.27±1.55	ll.38il.66	0.523	10.76il.36	ll.21il.51	0.098	0.036	0.465
GT total dose^*^ (mIU/ML)	2968.58i850.88	3349.50i948.68	<0.001	3 00 2.6 7i644.76	3457.lli870.50	0.003	0.526	0.316
Peak E2^*^ (pg/mL)	2734.08±1757.38	2373.08±1525.09	0.075	3453.24i2150.67	3478.43i2735.33	0.610	0.050	0.008
Peak P^*^ (ng/mL)	1.34±0.69	1.32±0.81	0.542	0.9Ü0.43	1.08±0.56	0.246	<0.001	0.011
Oocytes retrieved^*^ (n)	18.66±12.42	14.95i8.88	0.017	13.37i8.52	13.17±10.49	0.531	0.007	0.033
MII oocytes^*^ (n)	14.67±10.29	ll.74i7.52	0.006	9.78i6.33	9.74±7.68	0.707	0.001	0.029
Maturaty rate(%)	77.96±4.32	78.00il8.74	0.985	75.3±20.45	74.95±74.95	0.928	0.304	0.287

Values are presented as mean (standard deviation) or number (%). A p value of <0.05 was considered significant.

* The *P* value was calculated after log transformation for normal distribution.

rFSH, recombinant follicle-stimulating hormone; hp-hMG, highly purified-human menopausal gonadotropin; BMI, body mass index; FSH, follicle-stimulating hormone; AFC, antral follicle count; GT, gonadotropins; E2, estradiol; P, progesterone; MII, metaphase II

Note: Standard reference range: FSH: 1-9.2 mIU/mL

A multivariate linear regression analysis (Table 4) showed a negative effect of basal FSH, low AFC, GT type (non-rFSH) on the number of retrieved and MII oocytes, while only GT type (rFSH) was positively correlated with the maturity rate. AFC and GT type (rFSH) were positively correlated with E2 levels. The protocol type (GnRH antagonist or PPOS) was not found to affect the various ovarian stimulation outcomes.

**Table 4:** A Multivariate linear regression analysis for various ovarian stimulation outcomes.

Variable	Peak E2 (pg/mL)		Oocytes retrieved (n)		MII oocytes (n)		Maturaty rate(°/o)	
	Standardized Coefficient	*p* value	Standardized Coefficient	*p* value	Standardized Coefficient	*p* value	Standardized Coefficient	*p* value
Age(y)	-0.044	0.406	-0.061	0.176	-0.085	0.06	-0.033	0.535
Basal FSH (mUI/mL)	-0.056	0.288	-0.182	<0.001	-0.126	0.005	0.059	0277
AFC (n)	0.465	<0.001	0.451	<0.001	0.450	<0.001	0.054	0.330
Protocol type	-0.025	0.625	0.032	0.457	0.040	0.364	-0.020	0.705
GT type (rFSH versus non-rFSH)	-0.183	<0.001	0.095	0.032	0.136	0.002	0.113	0.033

*p* value of < 0.05 was considered significant.

E2, estradiol; MII, metaphase II; FSH, follicle-stimulating hormone; AFC, antral follicle count; GT, gonadotropins; rFSH, recombinant follicle-stimulating hormone.

Table 5 demonstrates a comparison between the rFSH group and the hp-HMG group only among 383 women who were treated with GnRH antagonist protocol. No significant differences in the age of the women (*p*=0.098) and the basal FSH (*p*=0.098) between the two groups were observed. The AFC was significantly higher in the rFSH group compared to the hp-HMG group (13.6±6.02 *vs*. 12.11±6.25; *p*=0.021). No significant differences between the rFSH group compared to the hp-HMG group were found in the total FSH dosage (*p*=0.375) or the duration of gonadotrophins treatment (*p*=0.376). The peak E2 level was significantly lower in the rFSH group compared to the hpHMG group (2506.93±1644.33 *vs*. 3631.45±2601.31 pg/ mL; *p*<0.001), while the peak progesterone level was significantly higher in the rFSH group compared to the hphMG group (1.30±0.75 *vs*. 1.04±0.53 ng/mL; *p*=0.013). The numbers of MII oocytes were significantly higher in patients stimulated with rFSH compared to those stimulated with hp-hMG (13.20±9.32 *vs*. 10.33±7.65; *p*=0.019), while the number of retrieved oocytes (16.81±11.23 *vs*. 14.13±10.38; *p*=0.055) and oocyte maturity rates (78.03±17.14 *vs*. 74.24±22.9; *p*=0.057) were at the limit of statistical significance.

**Table 5:** Comparison of ovarian stimulation data and outcomes between patients undergoing stimulation with rFSH or hphMG among women treated with the GnRH antagonist protocol^*^

		rFSH (n=296)			hp-hMG (n=87)	p value	
	Age, y	35.53 (2.09)			35.95 (2)	0.098	
	BMI (kg/m^2^)	23.32 (3.83)			23.33 (4.49)	0.995	
	Basal FSH (mIU/mL)	8.08 (2.82)			8.75 (3.92)	0.098	
	^†^AFC (n)	13.6 (6.02)			12.11 (6.25)	0.021	
	Ovarian stimulation duration (days)	11.29 (1.61)			11.08 (1.53)	0.376	
	^†^GT total dose (mIU/mL)	3170.05 (919.54)			3317.16 (835.58)	0.375	
	^†^Peak E2 (pg/mL)	2506.93 (1644.33)			3631.45 (2601.31)	<0.001	
	^†^Peak P (ng/mL)	1.30 (0.75)			1.04 (0.53)	0.013	
	^†^Oocytes retrieved (n)	16.81 (11.23)			14.13 (10.38)	0.055	
	^†^MII oocytes (n)	13.20 (9.32)			10.33 (7.65)	0.019	
	Maturity rate (%)	78.03 (17.14)			74.24 (22.09)	0.057	

* After ANCOVA with antral follicle count (AFC) as a covariate.

Values are presented as mean (standard deviation) or number (%). A p value of < 0.05 was considered significant.

† The *p* value was calculated after log transformation for normal distribution.

rFSH, recombinant follicle-stimulating hormone; hp-hMG, highly purified-human menopausal gonadotropin; GnRH, gonadotropin-releasing hormone; BMI, body mass index; FSH, follicle-stimulating hormone; AFC, antral follicle count; GT, gonadotropins; E2, estradiol; P, progesterone; MII, metaphase II

Note: Standard reference ranges: FSH: 1-9.2 mIU/mL

## DISCUSSION

The present study is the first to compare rFSH with hp-HMG for controlled ovarian hyperstimulation following an GnRH antagonist and a PPOS protocols in the setting of first-cycle elective FP. The findings demonstrated that rFSH yielded a higher number of both retrieved and MII oocytes compared to hp-hMG, although the proportion of mature oocytes to the total number of retrieved oocytes was similar for the two groups.

Our results are in agreement with previous reports (Bosch *et al*., 2008; Ana *et al*., 2011; Devroey *et al*., 2012; Shavit *et al*., 2016; Witz *et al*., 2020) in which follicular recruitment and development appeared to be more pronounced with rFSH compared with hp-hMG in combination with a GnRH antagonist. These outcomes may be explained by the potency of rFSH being higher than FSH isoforms in hp-hMG, resulting in the retrieval of significantly more follicles and oocytes. Indeed, basic FSH isoforms, such as rFSH, have a higher receptor affinity in vitro compared to more acid isoforms. Several studies suggested that given the higher biopotency in vitro, rFSH might also be more potent in vivo (Barrios-De-Tomasi *et al*., 2002; Filicori *et al*., 2002a; Andersen *et al*., 2004, 2011). This possible outcome could also be explained by the potential atretic effect on non-dominant follicles attributed to the LH/hCG effect (Hillier, 1994; Filicori *et al*., 2002b; Platteau *et al*., 2006). Nevertheless, we observed that hp-hMG treatment resulted in a similar maturity rate as that achieved with rFSH.

Despite the significantly lower oocyte number retrieved with hp-hMG, the E2 levels were substantially higher after treatment with hp-hMG compared with FSH. This finding has already been reported in GnRH antagonist cycles (Bosch *et al*., 2008; Ana *et al*., 2011; Devroey *et al*., 2012) and is attributed to the continuous exposure of follicles to the LH activity in the hp-hMG protocol, which induces higher levels of aromatizable androgens leading to higher E2 concentrations in the second half of the follicular phase (Smitz *et al*., 2007). Alternatively, the difference in E2 levels could be explained by different elimination kinetics of the FSH isoforms in the two gonadotropins preparations. On the other hand, serum progesterone concentrations were significantly higher in the rFSH group, a finding that had been already related to the administration of FSH in GnRH antagonist cycles (Bosch *et al*., 2008). This increase was explained by the stimulation of granulosa cell activity by FSH (Bosch *et al*., 2008; Filicori *et al*., 2002b; Andersen *et al*., 2006; Bosch *et al*., 2010), while LH may induce progesterone catabolism to androgens at the level of theca cells. Even minor elevation in progesterone levels at the end of stimulation negatively affects the endometrium and therefore implantation and ongoing pregnancy rates (Andersen *et al*., 2006; Bosch *et al*., 2010).

In the current study, we included two different ovarian stimulation protocols (GnRH antagonist or PPOS) with unequal distribution between the groups. Progestin for pituitary suppression during ovarian stimulation was reported to be an equivalent alternative to the GnRH antagonist protocol in women undergoing FP (Ata *et al*., 2021; Guan *et al*., 2021). This protocol has gained considerable popularity, making it essential to compare the ovarian response outcomes with different gonadotropins when following the PPOS protocol. We found no correlation between the protocol type and the ovarian stimulation outcomes in our current study, which included a small number of participants who had been treated with the PPOS protocol. Larger studies to confirm our preliminary findings are needed. In addition, when the rFSH and the hp-hMG groups were compared only among women treated with the antagonist protocol, similar results were found regarding the peak E2 levels, the peak progesterone levels, and the number of MII oocytes. The numbers of retrieved oocytes and oocyte maturity rates were higher in the rFSH group compared to the hp-hMG group, but the results were at the limit of statistical significance because of the relatively small number of participants.

The results of this study could have been affected by the clinical parameters between the two study groups having been significantly different (including the women’s age and AFC). Indeed, there was a negative effect of basal FSH and low AFC on the numbers of the retrieved oocytes and of the MII oocytes in a multivariate linear regression analysis. ANCOVA was performed (detailed in Methods) in order to overcome the obstacle of the difference in the patients’ AFC. Despite the significance between the women’s age, the difference in the average age of the women was extremely small (35.50 years and 35.99 years). To further explore this issue, we ran a subgroup analysis between patients younger than 35 years of age and patients 35 years of age or older and found no age difference within each subgroup. Both the younger and older patients in the rFSH group had better cycle outcomes compared to those in the hp-hMG group in terms of the number of retrieved oocytes and the number of MII oocytes. Given the above-described differences, however, the results should be viewed with caution. In the age-stratified analysis, the younger women in the rFSH group presented superior results regarding oocyte yield, as expected. However, the hp-hMG group presented unexpected resultsthe oocyte yield was similar in both age groups. A possible explanation for this may be that patients under 35 years using hp-hMG had another variable not contemplated by this study.

Importantly, all of the above-cited studies that compared the outcome of rFSH and hp-hMG were performed on infertile women undergoing treatment for IVF. Several differences that might affect ovarian response and future pregnancy outcomes should be considered in the setting of FP:

1) The frozen oocytes will be fertilized later by means of intracytoplasmic sperm injection (ICSI). Previous studies could not identify any significant differences in the fertilization rates, pregnancy rates, and pregnancy outcomes between patients treated with rFSH or hp-hMG in ICSI cycles (Bosch *et al*., 2008; Kilani *et al*., 2003; Platteau *et al*., 2004). One explanation could involve the interaction between oocytes and cumulus cells after oocyte retrieval that occurs only in IVF cycles. It has been proposed that the beneficial effect of the exogenous LH activity that is associated with the use of menotropins from the start of ovarian stimulation may materialize during the first hours of insemination in the IVF procedure and may be related to an enhanced effect of the cumulus cells on the oocytes resulting from increased LH activity in the stimulation period when menotropins are administered.

2) The parameter of the supraphysiological environment created by ovarian stimulation protocols that may be detrimental to both embryo implantation and placentation does not exist in FP.

3) The effect of the various gonadotropins on the endometrium does not affect future pregnancy outcomes.

Finally, as in most countries, the number of elective FP cycles is constantly increasing in Israel (Research and Information Center, State of Israel, 2021). Women and physicians must consider that the procedure is not funded, and therefore the total price of ovarian stimulation medications per cycle should be taken into account.

The present study has several limitations: (1) Its retrospective design limits the ability to obtain more details about factors that are known to impact fertility, including additional ovarian reserve markers, such as anti-Müllerian hormone and antral follicle count. (2) The statistical analyses were performed on small groups, which could compromise the strength of the conclusions. Further studies are needed to establish clear-cut conclusions. (3) The presented study provides a comparison on two different levels, specifically, on controlled ovarian stimulation level (GnRH antagonist or PPOS) and on ovarian stimulation (rFSH over hp-hMG), making it difficult to arrive at firm conclusions. (4) A difference in the final maturation trigger type proportion between groups can influence the results. (5) A very crucial limitation lies in the clinical differences of the groups of women, including age, AFC, and different control ovarian stimulations protocols, all of which can affect the results. Matched groups or prospective studies will eliminate these differences. (6) Prescribing gonadotropins according to the physician’s preference can introduce bias. (7) The current study lacks critical data pertaining to the use of oocytes, the fertilization rates, the rates of clinical pregnancies, and live birth outcomes. The omission of these pivotal metrics renders the weight of the evidence somewhat tenuous. That information awaits the findings of further studies. (8) There is no comparison between rFSH and a combination of rFSH and rLH.

## CONCLUSION

rFSH yielded a higher number of both retrieved and MII oocytes compared to hp-hMG when used in ovarian stimulation with GnRH and PPOS protocols for women undergoing elective FP. However, these findings should be treated with caution due to the possible influence of the differences between the groups in patient’s age, basal FSH, AFC and ovarian stimulation protocol. Further studies are required to explore the pregnancy and birth outcomes with diverse gonadotropins among women who undergo elective FP. Additional factors, such as availability, convenience, cost, and patient preferences should also be considered when choosing gonadotropins.
